# Editorial: Emotions and perception in cancer patients and survivors: the role of body image

**DOI:** 10.3389/fpsyg.2024.1434357

**Published:** 2024-06-05

**Authors:** Valeria Sebri, Silvia Francesca Maria Pizzoli, Davide Mazzoni, Jennifer Brunet, Csaba László Dégi, Gabriella Pravettoni

**Affiliations:** ^1^Applied Research Division for Cognitive and Psychological Science, IEO European Institute of Oncology IRCCS, Milan, Italy; ^2^Department of Psychology, Catholic University of Sacred Heart, Milan, Italy; ^3^Department of Oncology and Hemato-Oncology, University of Milan, Milan, Italy; ^4^School of Human Kinetics, University of Ottawa, Ottawa, ON, Canada; ^5^Department of Sociology and Social Work, Babeş-Bolyai University, Cluj Napoca, Romania

**Keywords:** body image, breast cancer, virtual reality, emotion, body

Body Image (BI) refers to the mental representation of the body and its related emotions within the bodily self-construct (Lewis-Smith et al., 2016; Sebri et al., 2021). It is a complex construct to research because it can be both positive or negative and because it encompasses various dimensions, including sensations, emotions, cognitions, perceptions, attitudes, and behaviors related to the body (Cash and Smolak, 2011), in particular to its *appearance* and *function*. Nonetheless, research on BI is important due to its influence on quality of life and its role in shaping social interactions.

Cancer can be a risk factor for the development of negative body image. Aesthetical changes after treatments can profoundly affect how individuals perceive their bodies, especially in terms of femininity and sexuality (Durosini et al., 2021). According to self-objectification theories (Fredrickson and Roberts, 1997), women diagnosed with breast cancer tend to constantly compare themselves to cultural stereotypes of physical appearance, resulting in feelings of body dissatisfaction, emotional distress, and social isolation (Sebri et al., 2022).

Additionally, interoception, which refers to the mental representation of sensations from the body, is crucial after an oncological experience as it is central to one's sense of self. After a breast cancer diagnosis, women may experience heightened bodily sensations, particularly in the breasts, leading to interoceptive consequences (Paterson et al., 2016). However, research shows different interoception consequences. Some studies suggest that increased awareness of bodily sensations may lead to heightened anxiety (Humphris and Ozakinci, 2008); others suggest that interoception can help to regulate internal states and promote emotional regulation (Herbert and Pollatos, 2012).

Overall, research on BI is developing fast, which helps our understanding of a foundational building block of individuals' quality of life after a cancer diagnosis. The Research Topic entitled “*Emotions and perception in cancer patients and survivors: the role of body image*” in Frontiers in Psychology presents current research in this area, along with select novel psychological interventions aimed at alleviating BI concerns in individuals diagnosed with cancer. The Research Topic includes a collection of six papers that bring together diverse perspectives including the innovative use of virtual reality (VR) to promote interoceptive awareness in women diagnosed with breast cancer and the complex interaction between aspects central to BI—from psycho-cognitive processes, to emotions, and to physical struggles.

The first paper presents a study protocol by Sebri et al.. This study evaluates an innovative VR intervention aiming to improve interoception, emotional wellbeing, fear of cancer recurrence, and body perception among women who are treated for breast cancer (Pizzoli et al., 2019). By utilizing VR as a human-computer interface, the intervention seeks to enhance participants' understanding and management of bodily sensations, potentially leading to increased awareness, reduced negative emotions, and better symptom management, offering insights for implementing VR psychological interventions in the future.

Second, Ding et al. shed light on the connections between psycho-cognitive factors and physical health in adults who had received or were currently receiving treatment for cervical cancer. Highlighting a high prevalence of depression, this study links adverse treatment reactions (e.g., physical symptoms), neuroticism, and marital relations to depression. Neuroticism mediated the relationship between adverse reactions and depression, with varying associations based on quality of life and marital relations.

Third, Huang et al. interviewed patients undergoing chemotherapy to explore symptom experiences and influential factors. Nine themes were generated from semi-structured interviews with 13 participants; themes reflect characteristics of symptom experiences and factors influencing symptom experiences. In light of their findings, the authors underscored the need for healthcare professionals to pay attention to chemotherapy-related symptoms, improve doctor-patient communications, and offer social support. Thus, improving infrastructure (e.g., healthcare service) and patient care processes is fundamental.

Fourth, a descriptive-correlational study by Moniri et al. demonstrated the positive association between depression and perceived stress in adults undergoing treatment for various cancer types. The study also showed that eating problems play a mediating role in this association. The authors emphasized the relevance of targeted interventions aiming to enhance emotion regulation and self-compassion to address depression and perceived stress in individuals facing cancer-related challenges.

Fifth, Wang et al. conducted a cross-sectional study to explore the relationship between BI, dyadic coping, and post-traumatic growth among 154 women undergoing treatment for breast cancer. BI was negatively associated with dyadic coping and positively with post-traumatic growth. Notably, BI was indirectly associated with post-traumatic growth via dyadic coping. Support for the mediational role of dyadic coping highlights its potential role in buffering the negative impact BI has for women diagnosed with breast cancer.

Lastly, a pilot study by Hasnaoui et al. evaluated an innovative fencing intervention focused on improving the quality of life among women who underwent surgery for breast cancer. The findings support continued investigation of the intervention as its acceptability was suitable and results suggest a trend in the improvement of quality of life and other relevant outcomes.

In conclusion, the papers featured in this Research Topic advance our understanding of contemporary perspectives on BI and adverse experiences associated with cancer and related common treatments such as depression and quality of life, emphasizing not only the value of investigating appearance but the overall body's functionality, as well as the need for holistic, tailored interventions. While much research has focused on negative BI, there is a growing emphasis on fostering positive BI. The integration of bodily awareness, personal inner experiences, psycho-cognitive, and psycho-social factors is crucial, particularly among those living with and beyond cancer within future research and while implementing interventions. Addressing the challenges and opportunities in this field, it is imperative to incorporate these insights into clinical practices to enhance the wellbeing in this population.

## Author contributions

VS: Writing – original draft. SP: Writing – original draft. DM: Writing – original draft. JB: Writing – original draft. CD: Writing – original draft. GP: Writing – review & editing.

## References

[B1] Cash T. F. Smolak L. (2011). Body Image: A Handbook of Science, Practice, and Prevention. New York, NY: Guilford Press.

[B2] Durosini I. Triberti S. Sebri V. Giudice A. V. Guiddi P. Pravettoni G. (2021). Psychological benefits of a sport-based program for female cancer survivors: the role of social connections. Front. Psychol. 12:751077. 10.3389/fpsyg.2021.751077 34899491 PMC8664561

[B3] Fredrickson B. L. Roberts T. A. (1997). Objectification theory: toward understanding women's lived experiences and mental health risks. Psychol. Women Q. 21, 173–206. 10.1111/j.1471-6402.1997.tb00108.x

[B4] Herbert B. M. Pollatos O. (2012). The body in the mind: on the relationship between interoception and embodiment. Top. Cogn. Sci. 4, 692–704. 10.1111/j.1756-8765.2012.01189.x 22389201

[B5] Humphris G. Ozakinci G. (2008). The AFTER intervention: a structured psychological approach to reduce fears of recurrence in patients with head and neck cancer. Br. J. Health Psychol. 13, 223–230. 10.1348/135910708X283751 18492319

[B6] Lewis-Smith H. Diedrichs P. C. Rumsey N. Harcourt D. (2016). A systematic review of interventions on body image and disordered eating outcomes among women in midlife. Int. J. Eat. Disord. 49, 5–18. 10.1002/eat.22480 26607999

[B7] Paterson C. L. Lengacher C. A. Donovan K. A. Kip K. E. Tofthagen C. S. (2016). Body image in younger breast cancer survivors: a systematic review. Cancer Nurs. 39, E39–58. 10.1097/NCC.0000000000000251 25881807 PMC4607543

[B8] Pizzoli S. F. M. Triberti S. Monzani D. Mazzocco K. Kufel E. Porebiak M.. (2019). “Comparison of relaxation techniques in virtual reality for breast cancer patients,” in 2019 5th Experiment International Conference (Exp. at'19) (Funchal: IEEE), 348–351.

[B9] Sebri V. Durosini I. Mazzoni D. Pravettoni G. (2022). Breast cancer survivors' motivation to participate in a tailored physical and psychological intervention: a qualitative thematic analysis. Behav. Sci. 12:271. 10.3390/bs12080271 36004842 PMC9404874

[B10] Sebri V. Triberti S. Pravettoni G. (2021). The self's choice: priming attentional focus on bodily self-promotes loss frequency bias. Curr. Psychol. 42, 378–389. 10.1007/s12144-021-01400-8

